# Bacteriophage Xp10 anti-termination factor p7 induces forward translocation by host RNA polymerase

**DOI:** 10.1093/nar/gkv586

**Published:** 2015-06-01

**Authors:** Nikolay Zenkin, Konstantin Severinov, Yulia Yuzenkova

**Affiliations:** 1 Centre for Bacterial Cell Biology, Institute for Cell and Molecular Biosciences, Newcastle University, Baddiley-Clark Building, Richardson Road, Newcastle upon Tyne, NE2 4AX, UK; 2 Waksman Institute, Rutgers, the State University of New Jersey, Piscataway, NJ, 08854-8020, USA; 3 Skolkovo Institute of Science and Technology, Skolkovo,143025, Russia; 4 Institute of Molecular Genetics, Russian Academy of Sciences, Moscow,123182, Russia; 5 Institute of Gene Biology, Russian Academy of Sciences, Moscow, 119334, Russia

## Abstract

Regulation of transcription elongation is based on response of RNA polymerase (RNAP) to various pause signals and is modulated by various accessory factors. Here we report that a 7 kDa protein p7 encoded by bacteriophage Xp10 acts as an elongation processivity factor of RNAP of host bacterium *Xanthomonas oryzae*, a major rice pathogen. Our data suggest that p7 stabilizes the upstream DNA duplex of the elongation complex thus disfavouring backtracking and promoting forward translocated states of the elongation complex. The p7-induced ‘pushing’ of RNAP and modification of RNAP contacts with the upstream edge of the transcription bubble lead to read-through of various types of pauses and termination signals and generally increase transcription processivity and elongation rate, contributing for transcription of an extremely long late genes operon of Xp10. Forward translocation was observed earlier upon the binding of unrelated bacterial elongation factor NusG, suggesting that this may be a general pathway of regulation of transcription elongation.

## INTRODUCTION

Transcription of DNA is the first step of gene expression. All three phases of the transcription cycle—initiation, elongation and termination—are heavily regulated. Regulation of elongation and termination is based on the control of the response of RNA polymerase (RNAP) to various pausing signals. Pausing is determined by sequences of nucleic acids within the elongation complex (EC), which alter the configuration of components of the active centre (1), slow down translocation (2) or cause reverse movement of the EC, known as backtracking (3). The response to these signals can further be controlled by elongation factors. For example, the elongation factor NusA mediates intrinsic termination and stimulates transcription pausing (4,5). In contrast, NusG, a universally conserved protein in all living organisms, stimulates elongation (6). NusG was proposed to lock the RNAP β′ clamp in a closed conformation, thus stabilizing EC and enhancing the processivity of elongation (7). NusG was also shown to promote forward translocation of RNAP (8), which should also increase processivity by suppressing pre-translocated and backtracked pauses.

A number of factors regulating transcription elongation are used by bacteriophages to highjack RNAP of the host bacteria to serve their own needs. One of such proteins is a 7 kDa protein p7 of the Xp10 bacteriophage (9), which infects *Xanthomonas oryzae*, a rice pathogen that causes rice blight, one of the most important diseases of rice in most rice-growing countries. p7 shuts down the bulk of host transcription and participates in switching of the expression from early to the late set of phage genes (10,11). Late genes are arranged in an unusually long operon that is punctuated by multiple intrinsic terminator sequences. It has been suggested that p7 aids transcription of distal late Xp10 genes by attenuating termination and pausing (10), similarly to phage λ transcription antiterminator protein Q (5).

p7 interacts strongly with the very N-terminus of the RNAP β′ subunit (12). A weaker interaction of p7 with the flap domain of the RNAP β subunit was reported recently (13). It was suggested that p7 first binds the β′ N-terminus and then the complex undergoes a structural rearrangement, which enables p7 to bind to the β flap (13). Both binding sites are in close proximity to domains of RNAP interacting with the RNA–DNA hybrid, the upstream edge of the transcription bubble, and the upstream DNA duplex.

The shutdown of host transcription and transcription of early phage genes during Xp10 development is caused by inhibition of transcription initiation from the −10/−35 class of promoters by p7 (9). Prevention of open promoter complex formation was attributed to the locking of the σ subunit in an inactive conformation in which its region 4 is unable to productively contact the −35 promoter consensus element. Recently, it was shown that p7 may also cause dissociation of σ (13). It is possible that both pathways are parts of one mechanism.

In contrast to the wealth of information about p7 action during transcription initiation, the mechanism of the antitermination by p7 remains obscure. It was shown that *in vitro* p7 abolishes termination on two unrelated terminators, tR2 and tR’, differing in lengths of their stem-loop structures and U-rich tracks (9). This observation implied that some general features of the EC are modified by p7, at least during antitermination. Moreover, it was shown that p7 was able to counteract the increased pausing and termination caused by NusA either by outcompeting NusA for binding to RNAP, or by negating NusA-induced modifications of the EC (see Discussion). Here, we demonstrate that p7 acts as a processivity factor suppressing various pauses and increasing the overall rate of transcription. We show that this general effect is based on the ability of p7 to induce forward translocation of the EC, likely by facilitating the over-winding of the upstream DNA duplex.

## MATERIALS AND METHODS

### Proteins and reagents

**Figure 1.**
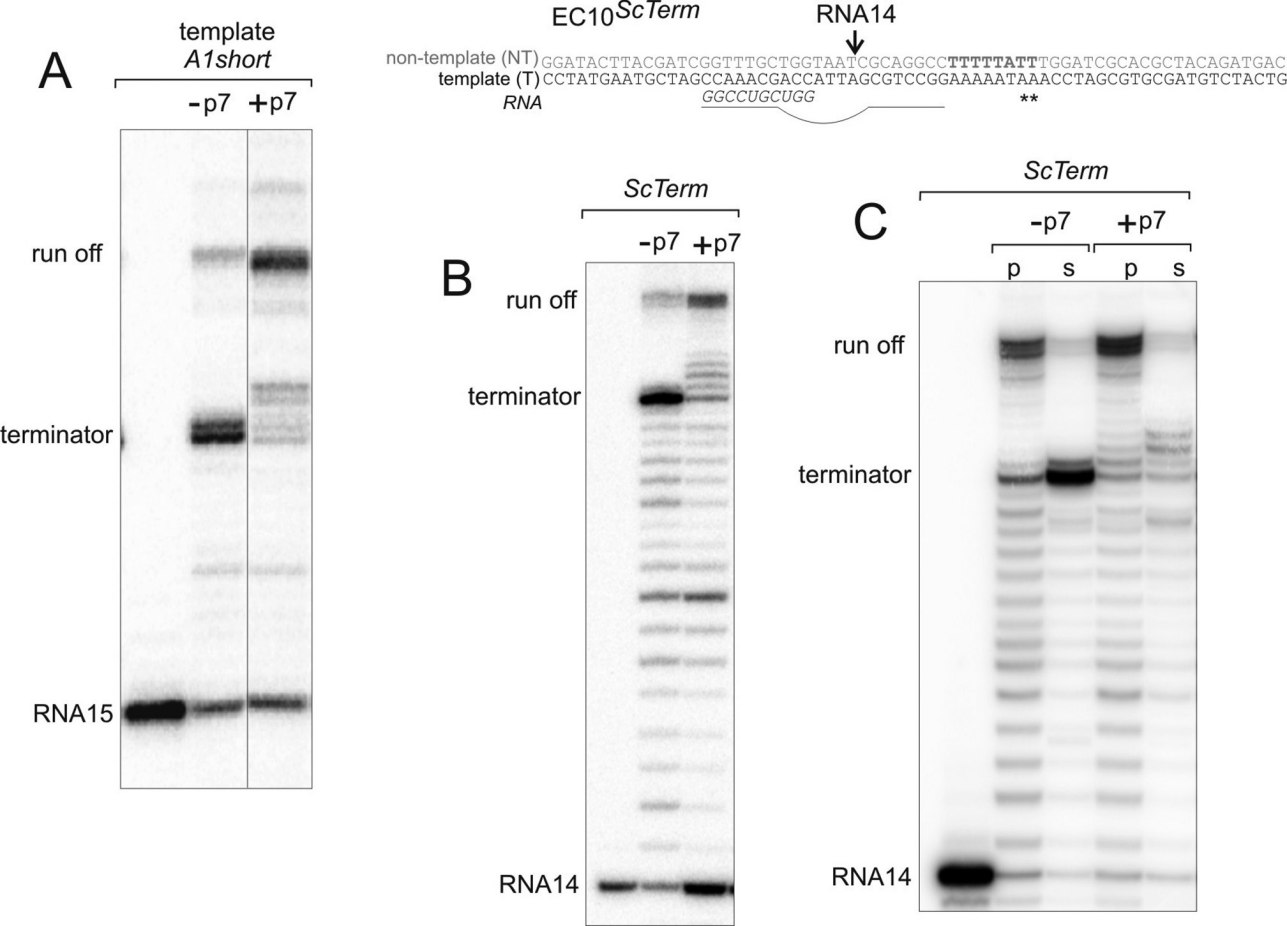
p7 induces antitermination and formation of longer termination products. (**A**) Chase (100 μM NTPs) of stalled elongation complex EC15*^A1short^*, obtained on *A1short* template containing tR2 terminator, in the presence or absence of p7. (**B**) Chase (100 μM NTPs) of EC14*^ScTerm^* obtained by walking assembled EC10*^ScTerm^* shown above the gel (region coding for termination hairpin is underlined; position of termination is indicated with asterisks), in the absence or presence of p7. (**C**) After chase of immobilized EC14*^ScTerm^* as in panel (B), reactions were divided into ‘supernatant’ and ‘pellet’ (ECs bound to the streptavidin beads which was further washed with TB) fractions. Note that Run off products are not released due to biotin on the 5′ end of template strand.

Native *X. oryzae* RNAP and recombinant hexahistidine tagged p7 protein of Xp10 phage were purified exactly as described (9). Radiolabelled nucleoside triphosphates (NTPs) were from Hartmann Analytic. Oligonucleotides were from Integrated DNA Technologies, Inc. The sequences of the templates and oligonucleotides of scaffolds used are shown in Supplementary Figure S1. The template DNA strands of assembled elongation complexes promoter-containing linear DNA templates were 5′ biotinylated (the reason why the run off products are not released in Figure 1C).

### *In vitro* transcription

All transcription experiments were done at 37°C in transcription buffer (TB) containing 20 mM Tris–HCl pH 7, 40 mM KCl, 10 mM MgCl_2_, unless otherwise specified. p7 was added where specified to 300 nM final concentration.

### Promoter initiated transcription

Open promoter complexes were formed by incubating 100 nM RNAP and 10 nM promoter-containing DNA for 15 min at 37°C. Stalled elongation complexes were obtained by using the following subsets of NTPs; EC11 on *A1long* template—50 μM primer CAUC, 20 μM adenosine triphosphate (ATP), 20 μCi of α[^32^P]GTP (3000Ci/mmol); EC20 on *A1long* template—50 μM primer CAUC, 20 μM ATP, CTP, 20 μCi of α[^32^P]GTP (3000Ci/mmol); EC37 on *IA347* template—50 μM CAUC, 20 μM ATP, CTP, 20 μCi α[^32^P]GTP (3000Ci/mmol); EC15 on *A1short* template 50 μM CAUC, 20 μM uridine triphosphate (UTP), cytidine triphosphate (CTP), 20 μCi α[^32^P]GTP (3000Ci/mmol). Stalled elongation complexes were immobilized on streptavidin beads, washed with TB containing 1M KCl and then with TB, and chased by addition of 100 μM NTPs for 10 min unless otherwise specified in figures. Reactions were stopped with formamide-containing buffer. Products were resolved by denaturing 15–23% polyacrylamide gelelectrophoresis (PAGE) (8 M Urea), revealed by PhosphorImaging (GE Healthcare) and analysed using ImageQuant (GE Healthcare) and SigmaPlot software (14).

### Transcription in the assembled EC

Artificial elongation complexes on scaffold *Sc1* and its derivatives were assembled and immobilized as described (15). Elongation complexes on scaffold *ScTerm* and its derivatives were assembled as described (16), except all complexes were immobilized on streptavidin agarose beads via biotin on the template strand. RNA in elongation complexes on scaffold *Sc1* and its derivatives was radiolabelled at 5′ end with γ[^32^P]ATP and T4 Polynucleotide kinase (PNK) (New England Biolabs) prior to complexes assembly. RNA in elongation complexes on scaffold *ScTerm* and its derivatives was radiolabelled during walking EC14*^ScTerm^* by incorporation of α[^32^P]UMP. EC14*^ScTerm^*, EC22*^ScTerm^*, EC16*^Sc1^* and EC14*^Sc1^* and their derivatives were obtained by walking with 10 μM NTP sets according to the sequence for 5 min and then were washed with TB. Reactions were initiated by addition of 100 μM NTPs or 500 μM PPi for 10 min, or left for hydrolysis for times specified in figures. Reactions were stopped and products analysed as above.

### Salt stability

To examine the stability of ECs, the streptavidin agarose beads containing bound ECs were incubated in TB containing 1 M KCl at 37°C for times specified in figures. Beads were pelleted, washed twice with 1 ml of TB and analysed as above.

### ExoIII footprinting

For ExoIII footprinting, the non-template strand of *A1long* was 5′ end-radiolabelled during polymerase chain reaction, the non-template oligonucleotides of scaffolds were 5′ end-radiolabelled before assembly. ECs obtained as above were treated with 100 units of ExoIII (New England Biolabs) 15 μl of TB for 10 min (*A1long*) or 1 (scaffolds) min. Reactions were stopped and analysed as above. Densitometry profiles in Figures were normalized to total radioactivity in each lane.

### KMnO_4_ footprinting in the assembled elongation complexes

ECs were 5′ end-radiolabelled at the non-template or the template strand, assembled and walked as above (where template strand was labelled, non-template was biotinylated). After washing they were treated with 10 mM KMnO_4_ at 37°C for 30 s. The reactions were stopped by adding β-mercapthoethanol to 1M and complexes were washed with ice-cold TB. DNA was cleaved with 10% piperidine for 20 min at 90°C. After the reactions cooled down, piperidine was extracted with chloroform (the beads were destroyed by the treatment and were left at the interphase). DNA was precipitated with ethanol in the presence of the calf thymus carrier DNA. DNA pellets were dissolved in formamide containing loading buffer and analysed as above. Densitometry profiles in Figures were normalized to total radioactivity in each lane.

### Electrophoretic mobility shift assay

DNA substrates were 5′ end-labelled with with γ[^32^P]ATP and PNK, and unincorporated γ[^32^P]ATP was removed by gel filtration on Micro-BioSpin 6 columns (BioRad). Radiolabelled DNA were mixed with single strand binding protein (SSB) (Sigma-Aldrich), p7 or the storage buffer, incubated for 10 min at 30°C and resolved by 6% native PAGE (0.5× TBE) at room temperature.

## RESULTS

### p7 alters a pattern of transcription termination products

We analysed single-round transcription by purified *X. oryzae* RNAP in the presence or absence of p7. Stable stalled elongation complexes, containing 15 nt-long RNA (EC15*^A1short^*) were formed on a linear DNA fragment *A1short* (for sequences of all templates and scaffolds used see Supplementary Figure S1), containing the T7 A1 promoter and a well-characterized tR2 terminator located between the transcription start-point and the transcription template end. EC15*^A1short^* were chased by the additions of NTPs in the absence or in the presence of p7. As expected, p7 strongly decreased transcription termination on the tR2 terminator (Figure 1A).

We performed a similar experiment with elongation complexes assembled on a nucleic acid scaffold containing the tR2 terminator sequence (Scaffold *ScTerm*; Figure 1B). Such scaffold complexes are an accepted model used for elucidation of molecular details of the termination process (16). Assembled elongation complexes are indistinguishable from those obtained by transcription from promoters, but allow much more freedom in manipulation of nucleic acids sequences in the EC (17). The initial complex, EC10*^ScTerm^*, was assembled using synthetic DNA and RNA oligonucleotides and *X. oryzae* RNAP. The RNA10 oligonucleotide was then extended and labelled in a template-directed fashion by incorporation of α[^32^P]NMPs to form EC14*^ScTerm^* (scheme in Figure 1B) (16). The complexes were next immobilized on streptavidin beads via a biotin molecule present in the template strand DNA oligonucleotide and washed from unincorporated nucleotide substrates. Upon chasing of the EC14*^ScTerm^* in the presence of NTPs, p7 readily caused antitermination (Figure 1B) in a very similar manner as on the promoter-containing template (Figure 1A), thus validating assembled complexes as an experimental system to investigate the mechanism of p7 action.

Note that, in the presence of p7, RNA products ending 1–5 nt downstream of the main termination site (seventh and eighth positions of poly-U signal) were formed (Figure 1A and B). Like the primary transcription termination product, these longer RNAs were true termination products as they were readily released from complexes immobilized on streptavidin beads through DNA (Figure 1C). Run-off products were not released because of the presence of streptavidin bead on the 5′ end of the template strand.

### p7 destabilizes EC and induces its forward translocation

**Figure 2.**
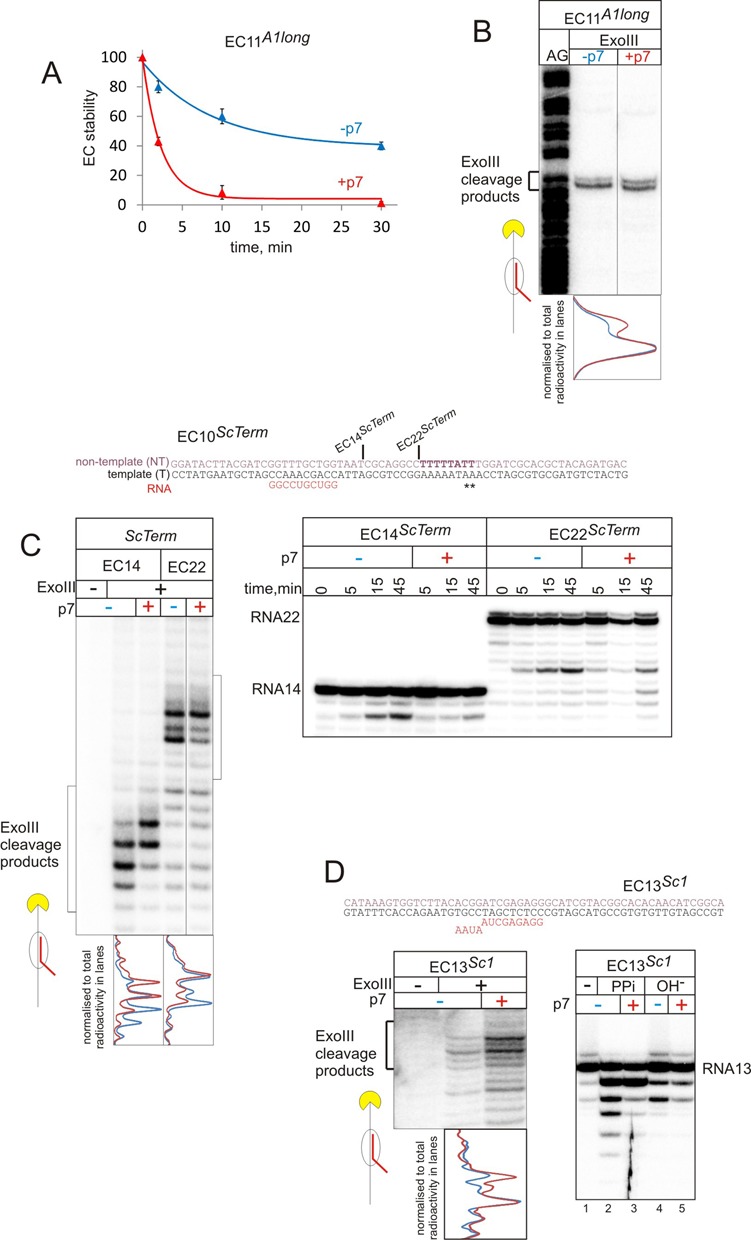
p7 destabilizes ECs and favours forward translocation of RNAP. (**A**) Stability of elongation complex with 11 nt-long transcript (EC11*^A1long^*) on *A1long* (see also Supplementary Figure S2 for stability of EC20*^A1long^*) After incubation in TB with 1M KCl in the absence (blue plot) or the presence of p7 (red plot) for the indicated periods of time, fractions of the ECs that remained on the beads after washing were measured. Data are mean and error bars are standard deviation from two independent experiments. (**B**) ExoIII footprinting of the front edge of RNAP in the EC11*^A1long^* formed on *A1long* template. Superimposed densitometry profiles of the lanes without p7 (blue) and with p7 (red) are shown below the gels. Here and after, profiles are normalized to total radioactivity in each lane. (**C**) ExoIII footprinting of the front edge of RNAP and kinetics of the intrinsic transcript hydrolysis in the absence or the presence of p7, in EC14*^ScTerm^* and EC22*^ScTerm^* formed on scaffold *ScTerm*. Superimposed densitometry profiles of the ExoIII footprinting without p7 (blue) and with p7 (red) are shown below the gels. Lesser signal in 15 min lane with p7 is due to loading defect. (**D**) ExoIII footprinting of the front edge of RNAP, intrinsic hydrolysis (30 min) and pyrophosphorolysis (10 min) in the absence or presence of p7, in EC13*^Sc1^* formed on scaffold *Sc1*. Superimposed densitometry profiles of the Exo III footprinting without p7 (blue) and with p7 (red) are shown below the gels.

Opening of the RNAP claw was proposed to happen at terminators, leading to EC disassembly and transcription termination (18,19). It is possible that p7 causes antitermination by stabilizing the ‘closed’ conformation of the RNAP crab claw, thus, in turn, stabilizing the entire elongation complex and preventing its dissociation to components (similar to the proposed mechanism of NusG function (7)). To test this idea, we measured the stability of the elongation complex EC11*^A1long^* obtained by T7 A1 promoter–driven transcription on DNA template *A1long*. The immobilized EC11*^A1long^* were washed after various times of incubation in transcription buffer containing 1 M KCl (high salt conditions were used to detect minor changes in EC stability), and the percentage of complexes that remained immobilized was determined and plotted (Figure 2A). Unexpectedly, p7 decreased stability of the elongation complex (Figure 2A). To test if proximity to the promoter of EC11*^A1long^* may have influenced response to p7 (though EC11*^A1long^* is known to be stable elongation complex that has already released σ-subunit (20)), we repeated the experiment with EC20*^A1long^* obtained on the same template. As can be seen from Supplementary Figure S2, p7 also destabilized EC20*^A1long^*. Furthermore, similar destabilizing effect of p7 was observed with elongation complexes assembled on nucleic acids scaffolds (below). Note that at low salt conditions (40 μM KCl) used in other experiments in this work, elongation complexes in the presence of p7 were stable (not shown). These observations suggest that destabilization of the elongation complex—apparently through a slight (seen only at higher salt conditions) change of contacts of RNAP with nucleic acids—is a characteristic property of p7 modification of the EC.

**Figure 3.**
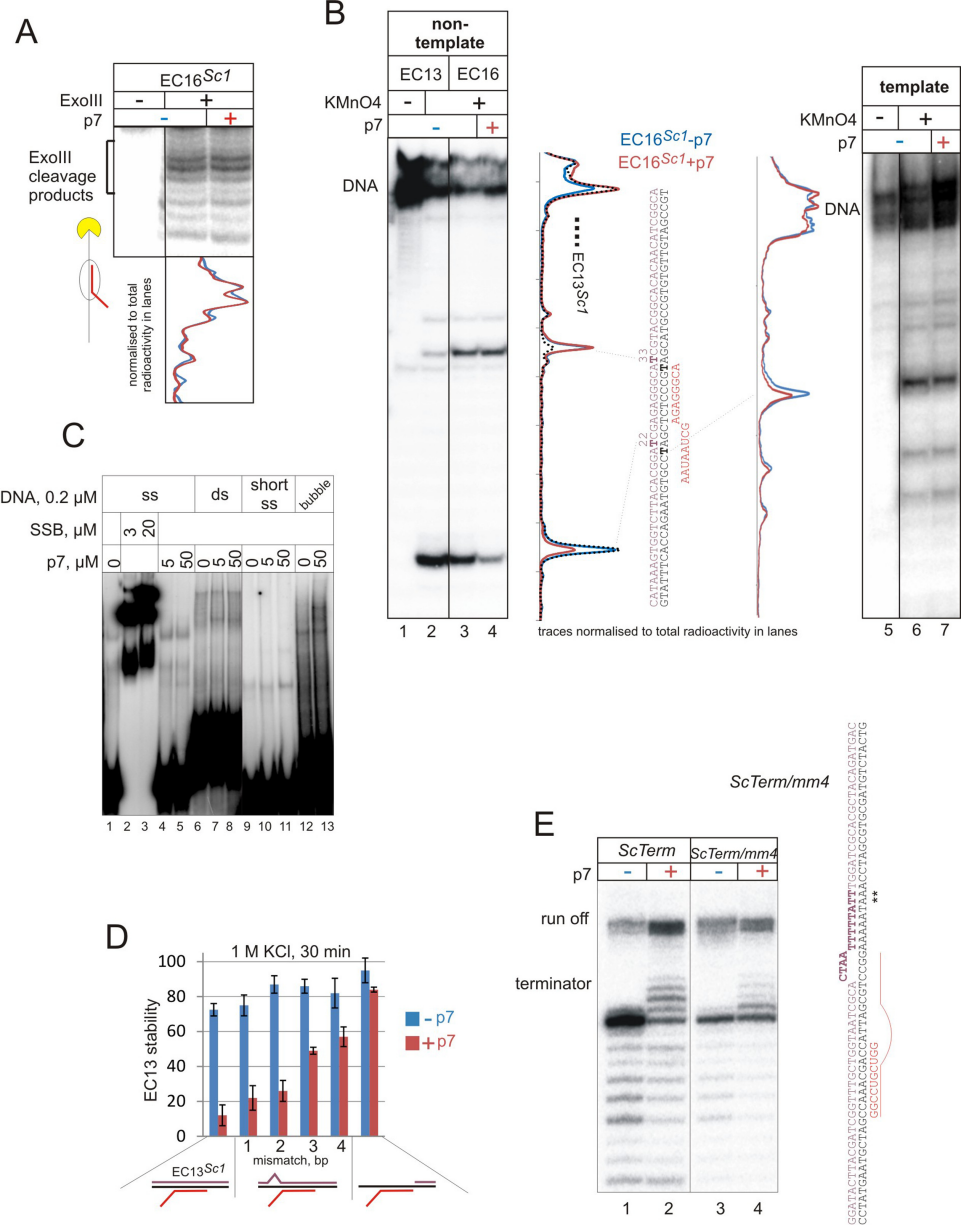
p7 induces more efficient rewinding of the upstream DNA duplex that results in bias towards more forward translocated complexes and destabilization of EC. (**A**) ExoIII footprinting of the front edge of RNAP in the EC16*^Sc1^* in the absence or presence of p7. Superimposed densitometry profiles of the lanes (normalized as above) without p7 (blue) and with p7 (red) are shown below the gel. (**B**) KMnO_4_ probing of elongation complex EC16*^Sc1^* in the absence or presence of p7 (probing of non-template strand in EC13*^Sc1^* is shown as a control for comparison of opening of T22 and T33; dashed line in densitometry profile). Non-template (left panel) or template (right panel) strand of the DNA was 5′ radiolabelled. Schemes of the complexes and superimposed densitometry profiles of the lanes without p7 (blue) and with p7 (red) are shown to the side of the gels. Profiles were normalized to total radioactivity in each lane in the gel. (**C**) EMSA of different DNA substrates in the absence or presence of increasing concentration of p7 (*Escherichia coli* SSB used as a positive control). (**D**) Stability of EC13 formed on scaffolds with the different state of the upstream DNA duplex; fully complementary template and non-template (*Sc1*), carrying mismatches of different lengths just upstream of the RNA–DNA hybrid, or lacking upstream portion of the non-template strand. The schemes of the assembled complexes are shown below the graph and sequences in Supplementary Figure S1. Measured is the fraction of ECs that remained bound to beads in the absence or the presence of p7 after 30 min incubation in TB with 1 M KCl and washing. Data are mean and error bars are standard deviation from three independent experiments. (**E**) Effect of mismatch in the upstream DNA duplex on termination pattern in the absence or the presence of p7. Transcription from EC14*^ScTerm^* with fully complementary template and non-template DNA strands and EC14*^ScTerm/mm4^* carrying 4 nt long mismatch just upstream of the RNA–DNA hybrid of the termination complex. The scheme of scaffold with mismatch is shown next to the gel, asterisks indicate termination positions.

The appearance of termination events downstream of the normal termination site in the presence of p7 (Figure 1A–C) suggests that p7 may induce forward translocation of the elongation complex. To test this possibility we analysed the position of the elongation complex using exonuclease III (ExoIII), which digests one of the strands of the double-stranded DNA in the 3′ to 5′ direction. Digestion of the non-template strand radiolabelled at the 5′ end thus reveals the distribution of positions of the front edge of the elongation complex on DNA. As can be seen from the ExoIII footprint presented in Figure 2B, p7 indeed induced a downstream shift of the front edge of EC11*^A1long^* (compare densitometry profiles below the gel). To test if the p7-induced forward shift of the elongation complex is a general phenomenon, we performed ExoIII footprinting of four additional ECs: EC14*^ScTerm^* and EC22*^ScTerm^* prepared by RNAP walking on Scaffold *ScTerm* (see scheme in Figure 2C), and EC13*^Sc1^* and EC16*^Sc1^* formed on an unrelated Scaffold *Sc1* (see scheme in Figure 2D). As can be seen from Figure 2C and D, p7 induced significant forward translocation of EC14*^ScTerm^*, EC22*^ScTerm^* and EC13*^Sc1^*. Importantly, p7 caused a bias towards more forward translocated states within the existing natural distribution of translocation states. In other words, p7 does not physically push the elongation complex but rather disfavours its backtracked states. The distribution of positions of EC16*^Sc1^* was not strongly affected by p7 (Figure 3A), probably suggesting that EC16*^Sc1^* does not frequently occupy backtracked states of its translocation equilibrium.

As ExoIII footprint may be influenced by overall change in the structure of the elongation complex, we tested the proposed p7-induced forward shift in the translocation equilibrium by analysis of hydrolysis of the nascent RNA. RNAP active centre can hydrolyze phosphodiester bonds of the transcript only in the pre-translocated or backtracked states, meaning that the rate of the reaction directly depends on the translocation equilibrium of the elongation complex. If p7 indeed facilitates forward shift of the EC, it must negatively affect the kinetics of hydrolysis. In full agreement with this prediction, RNA cleavage in EC14*^ScTerm^*, EC22*^ScTerm^* and EC13*^Sc1^* was slowed down in the presence of p7 (right panels in Figure 2C and D).

### p7 increases rewinding of the upstream DNA duplex

Because shifts between the translocation states of the elongation complex involve breakage and formation of DNA base pairs at the edges of the transcription bubble, the translocation equilibrium is very sensitive to the exact boundaries of the bubble (2). As p7 likely changes contacts of RNAP with nucleic acids (see above salt stability experiments), we hypothesized that it may affect the upstream edge of the transcription bubble. To test this, KMnO_4_ probing, which reveals unpaired thymidine bases in DNA, can be used. However, the forward shift of the elongation complex, in any case would be accompanied by apparent rewinding of the upstream DNA duplex, irrespective of whether this rewinding is the *cause* or a *consequence* of the shift. Therefore, to analyse the effects of p7 on the upstream edge of the transcription bubble, we used EC16*^Sc1^*, in which p7 had little effect on the translocation equilibrium (Figure 3A).

According to the accepted structure of bacterial elongation complex (2,21,22), base pair at position +33 (+1 relative the 3′ end of the transcript in EC16*^Sc1^*) is the most downstream base pair that can be melted in EC16*^Sc1^*. p7 did not change KMnO_4_ reactivity of T33 in the non-template strands of EC16*^Sc1^* (Figure 3B), which is consistent with little or no effect of p7 on the position of the front edge of EC16*^Sc1^* (Figure 3A), and confirms that translocation equilibrium of EC16*^Sc1^* does not have a significant backtracked component (also supported by strong increase in KMnO_4_-reactivity of T33 upon conversion of EC13*^Sc1^* to EC16*^Sc1^*; compare dashed and blue lines in densitometry profiles in the left panel of Figure 3A). In contrast, T22 of the non-template strand and T23 of the template strand, located at the upstream edge of the transcription bubble (positions −11 and −12 relative to the 3′ end of RNA, respectively), appeared to be more protected from KMnO_4_ modification in the presence of p7. As p7 does not induce forward shift of EC16*^Sc1^*, the result suggests that p7 somehow *facilitates* rewinding of the upstream DNA duplex.

Single-stranded DNA can potentially be protected from KMnO_4_ modification by bound p7 (though protection of both strands is unlikely given small size of the protein, see also Discussion). We, therefore, analysed the ability of p7 to bind nucleic acids by gel mobility shift assay using several different DNA substrates—single stranded (full length and a 15 nt-long piece of non-template strand of Scaffold *Sc1*), double-stranded (*A1short*) and a ‘bubble’ template (*A1short* with unpaired DNA strands around the start site). As a positive control, we used the *Escherichia coli* Single-strand binding protein (SSB). As can be seen from Figure 3C, in contrast to SSB that readily bound single-stranded DNA, p7 did not interact with any of the probes. Note, however, that this cannot exclude the possibility that DNA-binding ability of p7 manifests itself only in the context of RNAP, as it is the case for NusG from some organisms (23) (see also ‘Discussion’ section).

The above results suggest that p7 may facilitate rewinding of the upstream DNA duplex, thus disfavouring backtracked ECs. The bias toward more forward translocated states of the elongation complex cannot explain the observed p7-induced destabilization of the elongation complex, because backtracked complexes are as stable as forward translocated ones (3). We hypothesized that destabilization of the EC by p7 may also be caused by the excessive rewinding of the upstream DNA. To test this prediction, 1–4 nt-long mismatches were introduced into DNA upstream the RNA–DNA hybrid in EC13*^Sc1^*. Additionally, we used an EC with only downstream portion of the non-template strand present. Stabilities of complexes were measured in the presence of 1M KCl.

As expected, mismatches or removal of the upstream part of the non-template strand had little effect on the stability of the elongation complexes in the absence of p7 (Figure 3D). Consistently with our observation with EC11*^A1long^* obtained by synthesis from promoter (Figure 2A), p7 destabilized EC13*^Sc1^* with fully complementary template and non-template strands (Figure 3D). However, the destabilizing effect of p7 was gradually weakened with the increase of the unpaired region in the upstream DNA duplex (Figure 3D). In the absence of upstream portion of the non-template strand, p7 did not affect stability of the EC (Figure 3D). As mentioned above, stronger backtracking (observed in mismatched ECs by ExoIII and RNA hydrolysis assays; not shown) cannot account for stabilization in the presence of p7, as complexes, on their own, had similar stabilities. The result suggests that binding of p7 may alter interactions of RNAP with the upstream edge of the transcription bubble, which leads to excessive rewinding of the DNA duplex leading to destabilization of EC.

The hypothesis that p7 facilitates the rewinding of the upstream DNA duplex predicts that introduction of mismatches in the upstream DNA duplex of the termination complex would decrease the antitermination effect of p7, and should also remove the characteristic longer termination products observed in p7 presence. To test this prediction, we modified scaffold *ScTerm* by introducing a 4 nt-long mismatch just upstream of the T-rich tract (*ScTerm/mm4*), a region that corresponds to the upstream DNA duplex in the terminating EC (scheme in Figure 3E). Consistently with previous studies (24), the mismatch on its own caused significant antitermination (Figure 3E). Addition of p7, however, did not lead to any further increase in antitermination efficiency (Figure 3E). The amount of p7-specific longer-than-normal termination products was also strongly reduced (Figure 3E). Taken together the data support a hypothesis that effects of p7 on the elongation complexes and termination are caused by the ability of p7 to facilitate rewinding of the upstream DNA duplex in the elongation complex.

### p7 is a processivity factor

**Figure 4.**
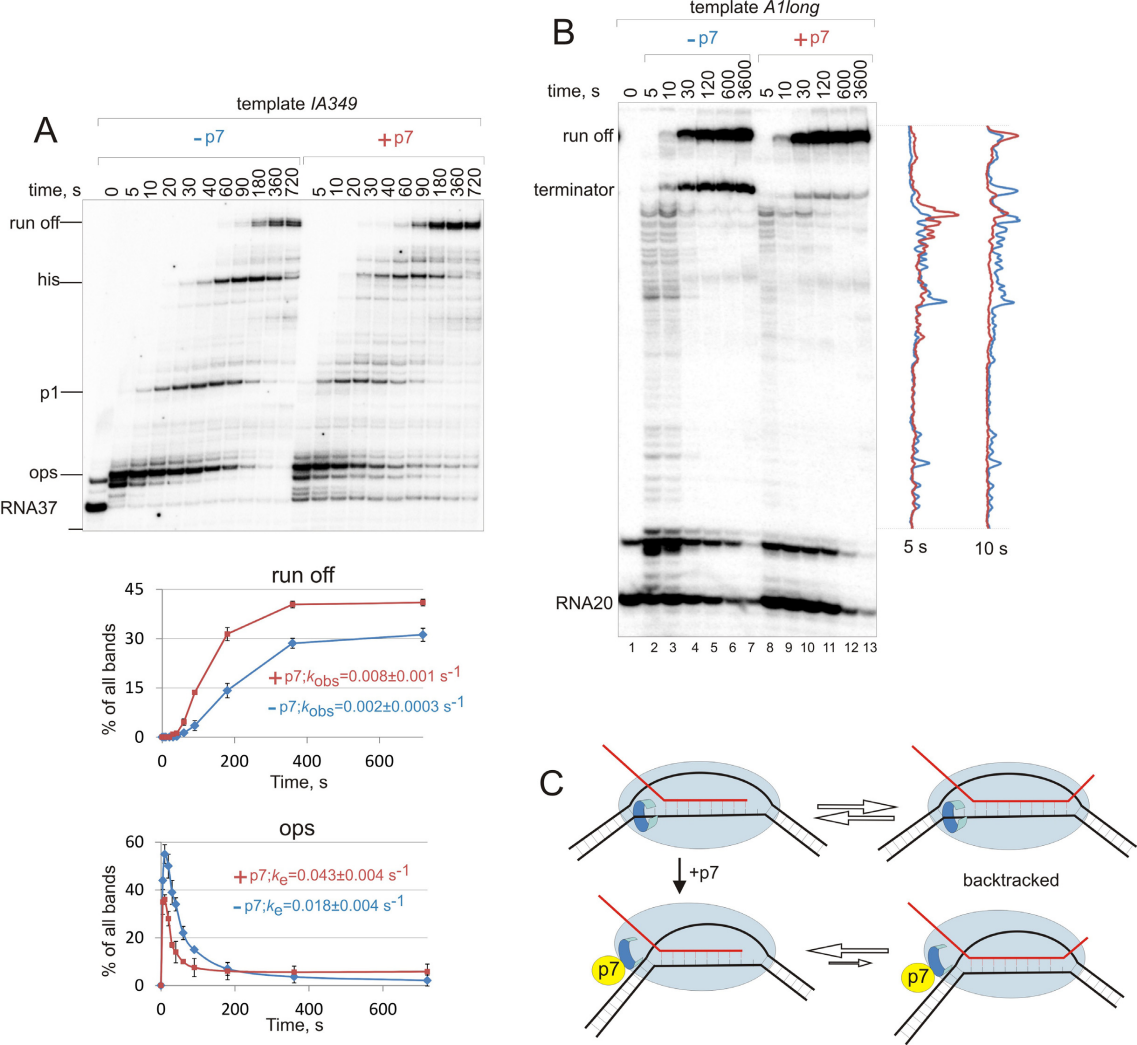
p7 is a processivity factor. (**A**) Chase to ‘run off’ (100 μM NTPs) of stalled elongation complex EC37*^IA349^*, obtained on the linear *IA349* template containing *ops, his* and uncharacterized pause *p1*, in the presence or absence of p7. Quantification of the bands for *ops* pause and run-off are presented below the gel (plots without p7 are blue and with p7 red). Data are mean and error bars are standard deviation from two independent experiments. Data were fit in single exponential equations and the predicted escape (*k*_e_) and growth (*k*_obs_) rate constants are shown next the plots (± sign is standard error of the fit). (**B**) Kinetics of chase (100 μM NTPs) of stalled elongation complex EC20*^A1long^*, on *A1long* template at the absence or presence of p7, densitometry traces from 5 and 10 s time point lanes are superimposed (traces without p7 are blue and with p7 red). (**C**) A model of p7 induced modification of the EC; our data suggest that p7 may perturb the contacts of RNAP with single-stranded region of DNA upstream of the RNA-DNA hybrid that leads to excessive rewinding of the upstream DNA duplex, which, in turn, (i) disfavours backtracking of EC, thus increasing processivity of transcription, (ii) weakens recognition of pause/termination signals and but (iii) decreases salt stability of EC.

The ability of p7 to bias translocation equilibrium towards more forward translocated states suggests that it may suppress pre-translocated and/or backtracked pauses during transcription elongation. The changes to the upstream part of the elongation complex facilitated by p7 may also decrease pauses caused by the structure of nucleic acids, such as hairpin-dependent pauses. In both cases p7 would be expected to increase processivity of transcription elongation, i.e. the overall speed of elongation and the success of overcoming pause signals. To test this possibility, we analysed kinetics of transcription elongation on two different DNA templates. The *IA349* template contained well-characterized *rfaQ ops* and *his* leader pause sites, as well as an uncharacterized pause ‘*p*1’ (25). As can be seen from Figure 4A, p7 decreased pausing at all three pause sites, allowing RNAP to reach the end of the template faster. A plot and constants in Figure 4A shows quantification of pausing at *ops* pause that has characteristics of pre-translocated and backtracked pause, which shows that both half-life and efficiency of pausing are reduced by p7. The presence of three pauses in one template complicates quantification of other two pauses. However in a very recent work p7 was shown to reduce both the half-life and efficiency of *his* pause (26). *T7A1long* template had a long stretch without major pauses. p7 facilitated faster elongation through this ‘pause-less’ sequence (Figure 4B; compare densitometry profiles).

## DISCUSSION

In this work we show that binding of bacteriophage Xp10 p7 protein shifts the translocation equilibrium of the EC towards more forward-translocated states, and thus increases processivity of transcription elongation by attenuating pausing and termination. Our data suggest that p7 promotes rewinding of the upstream DNA duplex in the EC and thus disfavours backtracking, which requires unwinding of the upstream DNA. Theoretically p7 could directly bind to the upstream DNA helix and stabilize it. Though p7 does not bind nucleic acids on its own, such activity could manifest itself in the context of the complex with RNAP, as was shown, for example, for RfaH, a transcription processivity factor and a NusG homologue (25). However, the binding site of p7 is at the very N-terminus of the β′ subunit (12), which is >70Å away from the upstream edge of the transcription bubble, a distance unlikely to be spanned by a 7 kDa protein. The p7 binding site is close to the RNAP ω subunit and the binding site for alarmone ppGpp (27,28). ω-dependent binding of ppGpp leads to alteration of the overall structure of the crab claw of RNAP. It is possible that binding of p7 near ω generates a similar allosteric signal that changes the structure of the EC and leads to rewinding of the upstream DNA. This proposal is supported by our demonstration that stability of the elongation complexes decreases upon p7 binding, which is indicative of a loss of some critical contacts between RNAP and nucleic acids. Furthermore, in strong support of this idea, a work that has been published while our manuscript was under review showed that antitermination activity of p7 depends on the ω subunit and can be modulated by ppGpp (26). The proposed changes to the overall structure and/or the observed changes to the contacts of RNAP with the nucleic acids in the EC may also alter recognition and/or stabilization of pauses. This could explain the reduction of pauses that are not caused by backtracking but rather by conformational changes in the EC (such as *his* pause).

We showed that mismatches in the upstream DNA duplex counteract p7-dependent destabilization of the EC and antitermination. Though the result could be explained by the fact that p7 can no longer interact with double-stranded upstream DNA, such direct interaction of p7 with upstream DNA duplex, as mentioned above, is unlikely. However, it is possible that alteration of the structure of RNAP upon p7 binding may destabilize contacts of RNAP with nucleic acids responsible for the maintenance of the upstream boundary of the transcription bubble. Upon binding at the N terminus of β′, p7 may affect the nearby β′ rudder, which interacts with the RNA–DNA hybrid and is one of the known determinants of the stability of the EC (29); the β′ lid which maintains the length of the RNA–DNA hybrid (30,31), and the β′ zipper that is close to, and may be involved in interaction with, the upstream edge of the transcription bubble and the upstream DNA duplex (32–34). We propose that p7-induced weakening of critical contacts that, during normal elongation inhibit the propagation of upstream DNA double helix further downstream, results in strengthening and/or excessive rewinding of the upstream DNA duplex (Figure 4C). This, in turn, disfavours backtracking, which must be accompanied by melting of the upstream DNA duplex and thus shifts translocation equilibrium of the EC towards more forward-translocated states (Figure 4C).

Besides shutting down transcription initiation from host and phage early promoters, p7 is needed to complete transcription of phage Xp10 late genes, organized in an extremely long operon that is longer than any of host bacteria operons and contains multiple pauses of different types and transcription terminators. Furthermore, p7 was very recently shown to act as a cofactor of NusA. NusA generally increases pausing and termination by RNAP, and thus would be highly harmful for development of phage such as Xp10. Surprisingly, p7 and NusA together result in even higher processivity and more efficient antitermination than achieved by p7 alone (26). As a processivity factor, p7 acts similarly to NusG (and its homologue RfaH), which increases processivity of RNAP to help it transcribe long operons, attenuating pausing and termination events. Interestingly NusG was also shown to favour forward translocation by RNAP (8), and thus relieve the pre-translocated and/or backtracked pauses such as the *ops* pause (2,4). NusG binds at the β′ clamp, relatively far from the p7 binding site, and, in contrast to p7, does not appear to destabilize the EC (7). This suggests that, although the mechanism of NusG-induced forward translocation remains obscure, it is likely different from that of p7. However, it also suggests that such mode of regulation of multi-subunit RNAP, while achieved by different ways, could be a common mechanism.

## SUPPLEMENTARY DATA

Supplementary Data are available at NAR Online.

SUPPLEMENTARY DATA
