# Supplementary material for: Bacteriophage Xp10 anti-termination factor p7 induces forward translocation by host RNA polymerase
Source: Nucleic Acids Res. 2015 Jun 1;43(13):6299–308. doi: 10.1093/nar/gkv586 (PMC4513864; doi:10.1093/nar/gkv586)
Supplement: SUPPLEMENTARY DATA [file supp_43_13_6299__index.html]

Bacteriophage Xp10 anti-termination factor p7 induces forward translocation by host RNA polymerase — Bacteriophage Xp10 anti-termination factor p7 induces forward translocation by host RNA polymerase — SUPPLEMENTARY DATA 

# Bacteriophage Xp10 anti-termination factor p7 induces forward translocation by host RNA polymerase

## SUPPLEMENTARY DATA

- SUPPLEMENTARY DATA
