# Supplementary material for: Bacteriophage Xp10 anti-termination factor p7 induces forward translocation by host RNA polymerase
Source: Nucleic Acids Res. 2015 Jun 1;43(13):6299–308. doi: 10.1093/nar/gkv586 (PMC4513864; doi:10.1093/nar/gkv586)
Supplement: SUPPLEMENTARY DATA [file supp_gkv586_nar-00325-m-2015-File006.pdf]

Supplementary Figures

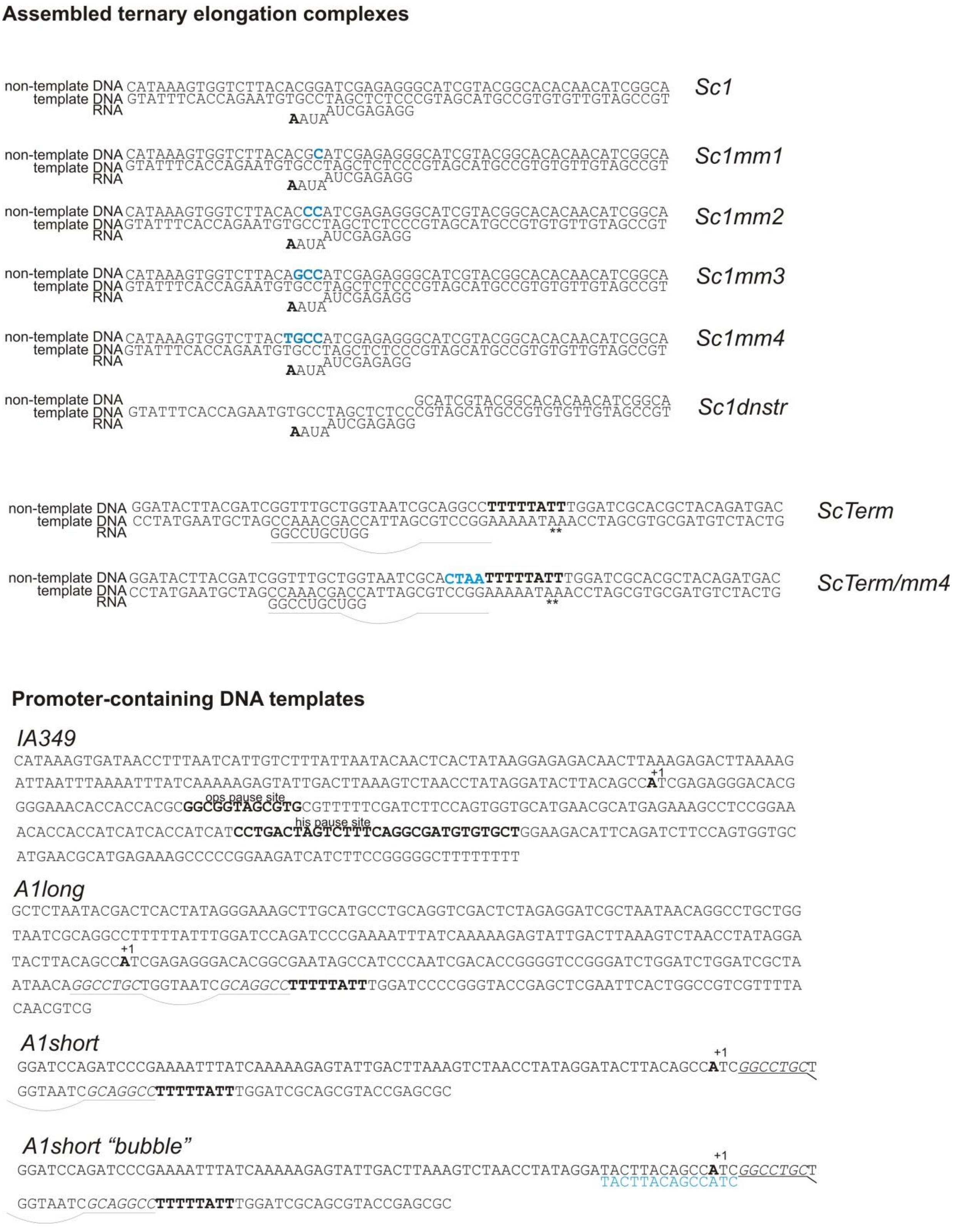

Figure S1. Templates used for this study. For assembled elongation complexes sequences of RNA, the non-template and the template DNA strands are shown. Mismatched bases are

shown in blue. For *ScTerm* and *ScTerm/mm4* the hairpin-coding region is underlined, U-rich terminating sequence is in bold, and termination positions are shown by asterisks.

For promoter-containing DNA only the non-template sequence is shown. Transcription start site is shown in bold. For *IA349* the *ops*-pause and *his*-pause sequences are shown in bold. For the *A1long*, *A1short* and *A1short* ”bubble”, the hairpin-coding region is underlined, U-rich sequence is in bold. Part of the template strand non-complementary to the non-template strand in *A1short”bubble”* is shown in blue.

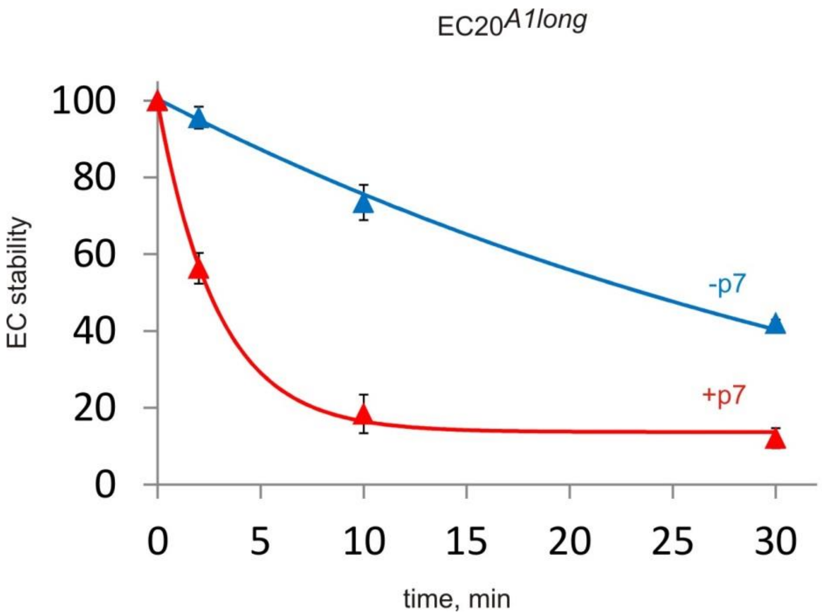

Figure S2. p7 destabilizes ECs. Stability of elongation complex with 20 nucleotide-long transcript (EC20^{A1long}) on *A1long*. After incubation in TB with 1M KCl in the absence (blue plot) or the presence of p7 (red plot) for the indicated periods of time, fractions of the ECs that remained on the beads after washing were measured. Data are mean and error bars are standard deviation from two independent experiments.
